# Resistance and Tolerance to Cryptococcal Infection: An Intricate Balance That Controls the Development of Disease

**DOI:** 10.3389/fimmu.2019.00066

**Published:** 2019-01-29

**Authors:** Mitra Shourian, Salman T. Qureshi

**Affiliations:** ^1^Translational Research in Respiratory Diseases Program, Meakins-Christie Laboratories, Research Institute of the McGill University Health Centre, Montreal, QC, Canada; ^2^Division of Experimental Medicine, Department of Medicine, McGill University Health Centre, Montreal, QC, Canada

**Keywords:** *Cryptococcus*, asymptomatic infection, damage response framework, disease tolerance, immunoregulation, host-pathogen interaction

## Abstract

*Cryptococcus neoformans* is a ubiquitous environmental yeast and a leading cause of invasive fungal infection in humans. The most recent estimate of global disease burden includes over 200,000 cases of cryptococcal meningitis each year. *Cryptococcus neoformans* expresses several virulence factors that may have originally evolved to protect against environmental threats, and human infection may be an unintended consequence of these acquired defenses. Traditionally, *C. neoformans* has been viewed as a purely opportunistic pathogen that targets severely immune compromised hosts; however, during the past decade the spectrum of susceptible individuals has grown considerably. In addition, the closely related strain *Cryptococcus gattii* has recently emerged in North America and preferentially targets individuals with intact immunity. In parallel to the changing epidemiology of cryptococcosis, an increasing role for host immunity in the pathogenesis of severe disease has been elucidated. Initially, the HIV/AIDS epidemic revealed the capacity of *C. neoformans* to cause host damage in the absence of adaptive immunity. Subsequently, the development and clinical implementation of highly active antiretroviral treatment (HAART) led to recognition of an immune reconstitution inflammatory syndrome (IRIS) in a subset of HIV+ individuals, demonstrating the pathological role of host immunity in disease. A post-infectious inflammatory syndrome (PIIRS) characterized by abnormal T cell-macrophage activation has also been documented in HIV-negative individuals following antifungal therapy. These novel clinical conditions illustrate the highly complex host-pathogen relationship that underlies severe cryptococcal disease and the intricate balance between tolerance and resistance that is necessary for effective resolution. In this article, we will review current knowledge of the interactions between cryptococci and mammalian hosts that result in a tolerant phenotype. Future investigations in this area have potential for translation into improved therapies for affected individuals.

## Introduction

The incidence of invasive fungal diseases has increased in recent decades and is associated with 1.5 million deaths annually. Much of this increase is attributable to the rising number of people with weakened or dysfunctional immune systems who are at high risk for the development of serious fungal infections (1–3). Major risk factors for invasive mycoses include HIV infection, stem cell, and solid organ transplantation, prolonged immunosuppressive therapy, invasive medical procedures, hematological malignancies, advanced age, and prematurity (4, 5). More than 90% of all reported fungal-related deaths result from species that belong to four genera: *Cryptococcus, Candida, Aspergillus*, and *Pneumocystis* (4). In addition to delays in diagnosis, similarities between eukaryotic fungi and humans render treatment of fungal infections more difficult compared to bacterial and viral infections. Relatively few antifungal drugs are currently available and their efficacy is limited by toxicity, a narrow spectrum of activity, detrimental drug interactions, the development of resistance, and, in some cases, high cost (6, 7).

The genus *Cryptococcus* contains at least 37 species; however, *C. neoformans* and *Cryptococcus gattii* are the main causes of human disease (8, 9). *Cryptococcus neoformans* classically targets immunosuppressed individuals including those with advanced HIV-AIDS, various T cell deficiencies, pregnancy, chronic lung, renal, or liver diseases, cancer, and patients receiving immunosuppressive therapy, while *C. gattii* has a predilection for immunocompetent individuals (10–12). The initial exposure to cryptococci occurs through inhalation of spores or small desiccated yeast cells that enter the lower respiratory tract. A seroprevalence study in New York demonstrated that 70% of samples from children over the age of 5 years had reactive antibodies against *C. neoformans* antigens, suggesting that exposure is widespread despite a low incidence of disease (13). Although definitive human studies are lacking, circumstantial evidence indicates that asymptomatic colonization of the airways or latent cryptococcal infection of the lungs and associated structures may also be common (14, 15). For example, autopsy studies identified *C. neoformans* infection in subpleural or parenchymal lung nodules where yeasts were contained inside macrophages and multinucleated giant cells in association with a granulomatous response (16–18). On the other hand, the most devastating clinical consequence of cryptococcal infection is meningoencephalitis that can occur following a primary lung infection or by reactivation and dissemination of latent pulmonary infection upon subsequent immunosuppression (19–21). The development of severe cryptococcal disease may occur years or even decades after the initial infection, indicating that humans are able to tolerate the presence of viable cryptococci for extended periods of time (22).

A recent study of the global burden of cryptococcal disease estimated that 278,000 individuals have a positive cryptococcal antigen test that is indicative of infection and 223,100 patients develop cryptococcal meningitis, with 73% of the cases occurring in Sub-Saharan Africa (23). Worldwide, cryptococcal meningitis account for 181,100 deaths annually, including 15% of AIDS-related deaths. These figures indicate that the proportion of AIDS-related mortality has not changed compared to the previous estimate in 2008 (24) with cryptococcosis remaining the second most common cause of AIDS-related death after tuberculosis (23). Notably, up to 20% of cases of cryptococcosis occur in phenotypically “normal” or apparently immunocompetent patients without any known risk factors for infection susceptibility (25). Almost 50% of patients with cryptococcal meningitis die in the year after infection mainly because of unsuccessful therapy (26). A better understanding of the key mechanisms of host immunity to *Cryptococcus* will be important for future development of new and more effective approaches to preventing and treating cryptococcal diseases. The mechanisms of host resistance in *Cryptococcus* infection has been extensively studied and reviewed elsewhere (20, 21, 27, 28). In this article, we will discuss the mechanisms of tolerance that characterize the host-cryptococcal interaction.

## Overview of Tolerance and Resistance

The concept of disease tolerance was originally described in plants and arose from observations of variation in disease severity at a population level without a direct correlation to pathogen load (29–31). Compared to resistance, which is defined as the ability to reduce pathogen burden to preserve homeostasis, tolerance is the ability to limit the extent of damage and dysfunction to host tissues during infection. Disease tolerance pathways that attempt to maintain host fitness without exerting direct negative effects on pathogen burden may lead to microbial survival and persistence (32–34).

Two types of tissue damage may occur during infection; one is directly caused by the pathogen through toxin production and virulence factor expression, and can be limited by reduction of the microbial load through host resistance mechanisms. The second type of tissue damage is an indirect consequence of infection that results from a vigorous host immune response and manifests as immunopathology despite control of pathogen burden (33). Certain host resistance mechanisms have potentially damaging effects on host fitness; for example, production of reactive oxygen species (ROS), proteases, and growth factors by neutrophils and macrophages may cause cellular destruction, abnormal collagen deposition, and tissue fibrosis (35). Even if overt organ damage is not evident, host resistance mechanisms are usually associated with some degree of subclinical tissue dysfunction; for example, inflammation that is effective in combating lung infection can alter both the integrity and permeability of the pulmonary vascular endothelium and airway epithelium and may culminate in reduced respiratory function (31).

In general, disease tolerance is characterized by stress responses and damage control mechanisms that maintain homeostasis and functional integrity of host tissues in response to environmental changes. When physiological parameters change beyond a certain threshold, stress responses initiate signal transduction pathways to provide metabolic adaptation in host cells (32). Some of the best known signaling mechanisms involved in the cellular stress responses include transcription factors such as HIF-1alpha (hypoxia-inducible factor 1 alpha) triggered by hypoxia, NRF2 (nuclear factor-erythroid 2-related factor 2) triggered by oxidative stress, and AhR (Aryl hydrocarbon Receptor) triggered by xenobiotic stress (36–38). Other stress response mediators include AMPK (AMP-activated protein kinase) triggered by ATP depletion, and the NLR (Nod-like receptor) protein family that responds to stress caused by microbial toxins and endogenous danger signals (33, 38). In a similar manner, tissue damage control can also occur through various mechanisms that (1) enforce barrier function of epithelial cells and prevent pathogen access to host tissue, (2) neutralize pathogen toxins and virulence factors, (3) regulate the intensity and duration of the host immune and inflammatory responses and (4) enhance resistance against inflammatory damage by promoting parenchymal cell regeneration (32, 33, 39).

**Figure 1:**
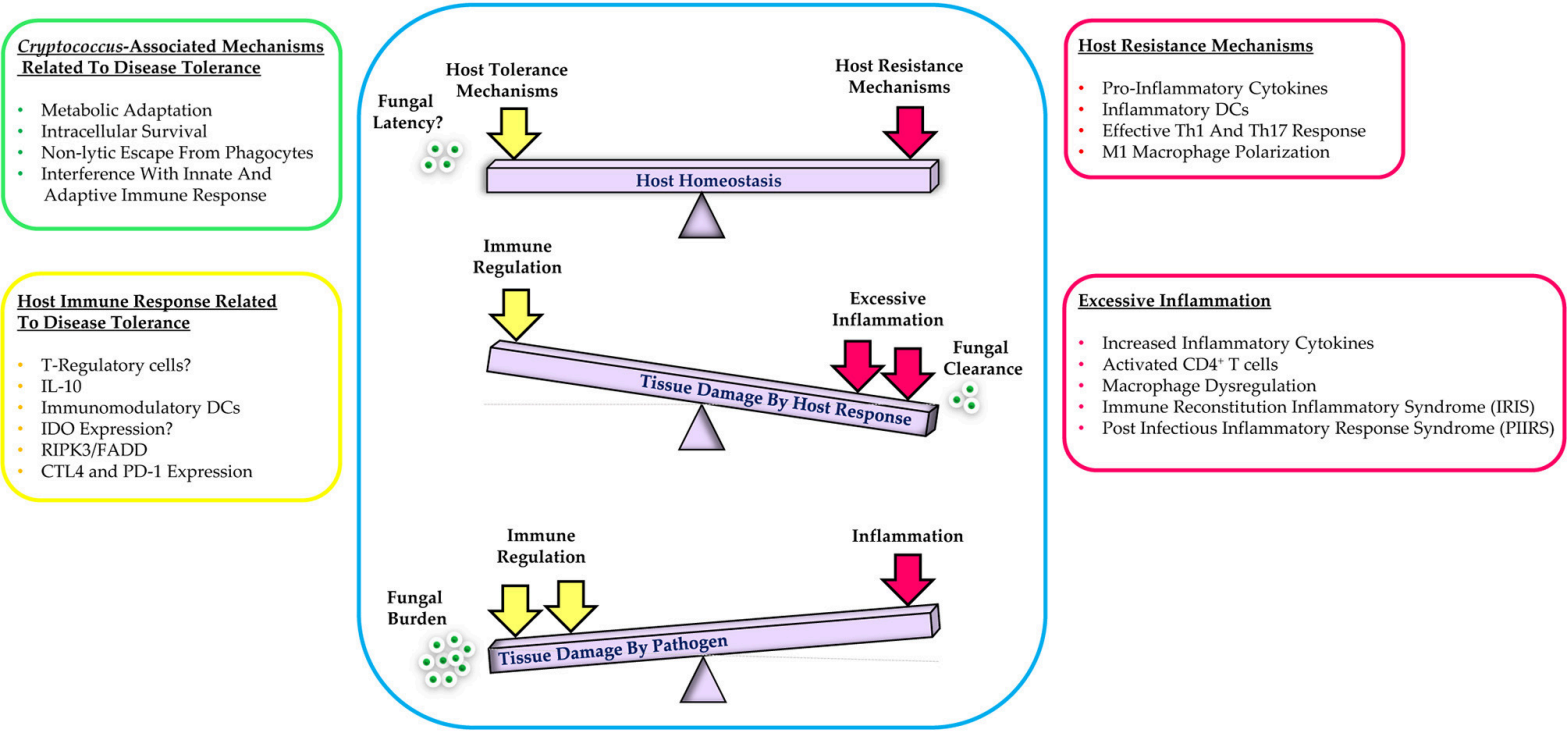
Schematic representation of the balance between host resistance and tolerance to cryptococcal infection. Effective control of infection requires a balanced response between host tolerance and resistance mechanisms while excessive host inflammation or immune regulation leads to tissue damage. Additional details are provided in the text.

Mechanisms of host resistance and disease tolerance function in a pathogen class-specific manner (33). In some cases, the pathogen itself may contribute and/or augment the host's capacity for tolerance to enhance its own survival and transmission. If the host can sustain a high level of tolerance that is sufficient to prevent major disruption of physiological functions, a state of persistent and/or asymptomatic infection will be established. Conversely, if host resistance mechanisms cause significant tissue damage or major alterations of host physiology, various pathological outcomes of infection will occur (31). Ultimately, an ideal immune response is defined by the balance between host resistance and tolerance that facilitates efficient pathogen clearance with an acceptable degree of immunopathology Figure 1 (32).

## Disease Tolerance and the Damage Response Framework in Host-*Cryptococcus* Interaction

Based on serological and epidemiological studies, natural exposure to *Cryptococcus sp*. is common. Yet, despite the observation that a high percentage of children and healthy individuals in certain geographic areas develop cryptococcal antibodies, overt clinical manifestations of disease are rare (13, 22, 40–42). In an immunocompetent host, infectious propagules of *Cryptococcus sp*. are completely cleared from the respiratory tract or may establish a latent asymptomatic infection in pulmonary granulomas or thoracic lymph nodes (15, 16). Following immunosuppression, the fungus can proliferate and disseminate to other parts of the body, including the central nervous system. Given the lack of an inflammatory response during latent infection, symptoms of disease reactivation will not develop until the fungal cell burden causes tissue dysfunction and damage to infected organs (8, 22, 43). Depending on host factors, cryptococci may cause progressive granulomatous inflammation or form discrete fungal masses (termed cryptococcomas) in primary target organs such as the lungs and brain. Each of these vital organ systems has a relatively low tolerance and repair capacity and is highly susceptible to damage; therefore, severe and/or progressive infection of the lower respiratory tract or central nervous system is poorly tolerated and life-threatening (31, 44). Indeed, latent asymptomatic cryptococcal infection, but not clearance, may be considered as a host tolerance mechanism to prevent or limit lung or brain damage (45).

The indispensable role of the host response to the outcome of microbial pathogenesis is a central tenet of the Damage Response Framework (DRF) proposed by Pirofski and Casadevall (46, 47). The DRF integrates the contribution of microbial and host factors that may produce a net benefit or cause disease that is reflected by host damage. Importantly, microbial virulence traits interact with either a weak or strong immune response to cause disease that exhibits a parabolic distribution. In addition to disease, the highly dynamic interaction between microbe and host may also lead to different disease outcomes including colonization, latency, and commensalism. From the viewpoint of the DRF, progressive asymptomatic cryptococcal infection will continue until the damage resulting from host-pathogen interactions over time exceeds a threshold amount that is sufficient to create clinical symptoms (47, 48). *Cryptococcus neoformans* has been classified as a class 2 pathogen that causes disease exclusively in hosts with weak or defective immune responses through expression of virulence traits. However, the emergence of *C. gattii* in apparently healthy individuals in Pacific Northwest and development of immune reconstitution inflammatory syndrome (IRIS)-associated cryptococcosis in HIV/AIDS after antiretroviral therapy, suggests that cryptococci may be class 4 pathogens that cause disease at the extremes of weak and robust immunity. Thus, the pathogenesis of cryptococcal disease and associated host damage is attributable to the interaction of fungal virulence with dysregulated host immune responses (47–49).

As reviewed elsewhere, protection against cryptococcal infection is mainly associated with secretion of pro-inflammatory cytokines, generation of effective Th1/Th17 adaptive immune responses, and classical activation of macrophages that mediate fungal clearance (20, 21, 27, 28, 50–53). Although resistance mechanisms are required for sterilizing immunity, excessive inflammation can be detrimental to the host and culminate in severe tissue damage and immunopathology. In fact, an ideal immune response to cryptococcal infection necessitates a tightly regulated balance between Th1, Th17, and Th2 responses that control fungal growth while preventing excessive tissue damage and immunopathology (Figure 1) (19, 21). The pathological consequences of excessive inflammation during cryptococcal infection are clearly exemplified by the problem of IRIS. Development of cryptococcal IRIS is mainly associated with HIV+ patients, solid organ transplant recipients, and pregnancy and is caused by recovery of specific immune responses resulting in exaggerated host inflammation and local organ damage (54). There are two types of cryptococcal IRIS in HIV+ patients: (1) Paradoxical cryptococcal IRIS that occurs after starting ART and presents as a deterioration or recurrence of clinical symptoms in the same or new site even with successful antifungal therapy, and (2) Unmasking cryptococcal IRIS that begins shortly after initiation of ART in patients with no prior diagnosis of cryptococcosis and may be its first manifestation (55–57). A paradoxical immune response, known as post-infectious inflammatory response syndrome (PIIRS), can also occur in non-HIV patients with cryptococcal meningitis following reduction of immunosuppressive therapy and is associated with severe neurological disease (58, 59).

In the context of the damage response framework, cryptococcal meningitis can be classified in 3 groups (44, 55): (1) In HIV+ patients who have not started highly active antiretroviral therapy, host damage is mainly pathogen-mediated and is characterized by a high fungal burden. Even after initiation of effective antifungal therapy, pathogen virulence is believed to be a major determinant of mortality. Low levels of Th1-associated cytokines including IFN-gamma and TNF-alpha in these patients suggest that immune-associated damage is not a major factor in disease pathogenesis (60, 61). These observations are also consistent with a lack of significant improvement in disease outcomes with adjunctive corticosteroid therapy (62). (2) In HIV+ patients that develop cryptococcal IRIS after starting the antiviral therapy, damage is associated with a vigorous Th1 type host immune response that is characterized by increased inflammatory cytokines IFN-γ and IL-6, activated macrophages/monocytes, and recruitment of CD4^+^ T cells. Induction of cerebral edema, neurotoxic effects of activated macrophages, and metabolic programming of neurons by adjacent inflammatory signals are some of the mechanisms of immune-mediated damage in the brain (63–65). (3) In non-HIV patients, tissue damage is mainly associated with a robust intrathecal Th1 type cellular immune response that is associated with alternative macrophage activation, high IL-10 and low TNF-α levels. The discordant activation of lymphocytes and macrophages results in persistent expression of cryptococcal antigen that perpetuates local inflammation (44, 55, 59). To maintain homeostasis and prevent unnecessary tissue damage, host tolerance mechanisms regulate the degree and duration of the immune response; therefore, the development of IRIS, a condition that is characterized by excessive and dysregulated immunity, could signify a failure of tolerance during cryptococcal infection (30, 66).

Excessive inflammation and immune-mediated host damage have also been shown in experimental mouse models of cryptococcal IRIS. Following CD4^+^ T cell transfer into RAG^−/−^ mice on the C57BL/6 or BALB/c genetic background, severe inflammatory disease was established in lungs, brain, and liver without affecting fungal clearance. Compared to controls, heightened systemic inflammation characterized by Th1-type cytokines and activated CD4^+^ T cells as well as granulomatous inflammation of the liver was observed in reconstituted RAG^−/−^ mice (67). In another model, C57BL/6 mice infected intravenously with 10^6^ *C. neoformans* 52D developed lethal neurological dysfunction 3 to 4 weeks post-infection despite fungal clearance in the central nervous system. Activated microglia and antigen-specific IFN-γ producing CD4^+^ T cells were identified in the brains of infected mice. Depletion of CD4^+^ T cells reduced CNS inflammation and prevented mortality, although fungal clearance was also decreased (68). Interestingly, despite an extremely high fungal burden at day 7 and 14 post-infection, the presence of central nervous system infection remained relatively asymptomatic. One explanation for this observation could be host tolerance to infection that was ultimately subverted by a vigorous immune response and the development of extensive tissue damage.

## *Cryptococcus*-Associated Mechanisms Related to Disease Tolerance

Microbial pathogens employ a variety of mechanisms to trigger host damage including intracellular and/or extracellular replication, production, and release of toxic substances, disruption of organ homeostasis, and modulation of host immune responses (47). *Cryptococcus sp*. express several virulence factors that facilitate pathogen survival, proliferation, and dissemination in mammalian hosts (69–71). The mechanisms by which *C. neoformans* mediates host damage have been extensively reviewed by Casadevall et al. (72). At the molecular level, *C. neoformans* produces several degradative enzymes such as proteases, urease, phospholipase, and nuclease that degrade host molecules (73–77). Mechanisms of cellular damage include: (1) interference with phagolysosome maturation (78), (2) increased permeability of the phagosome membrane (79, 80), (3) disrupted organelle function; for example, the ability to impair protein synthesis by mitochondria (81, 82), (4) cytoskeletal alterations (83), (5) non-lytic exocytosis and cytoplasmic vacuolation (84–86), and (6) lytic exocytosis resulting in host cell death (87). In addition, *C. neoformans* has several direct and indirect mechanisms that interfere with host immune cell function and damage endothelial cells in the brain vasculature (72).

In contrast to the virulence factors and microbial mechanisms that trigger cell and tissue damage as part of disease pathogenesis, *Cryptococcus sp*. has evolved several unique strategies that facilitate survival and persistence in the host without causing apparent pathology. Remarkably, the persistence of a chronic, low-grade *C. neoformans* infection does not prevent the generation a protective cell-mediated immune response upon secondary infection (47, 88). Some of the main strategies that contribute to latent cyptococcal infection and prevent complete clearance include acquisition of stress tolerance mechanisms against high temperature, reactive oxygen species, and reactive nitrogen species, capacity for facultative intracellular residence, regulation of host cell expulsion mechanisms, and evasion or interference with innate and adaptive immunity (19, 45, 89, 90). Below, several important characteristics associated with long-term or persistent cryptococcal infection are summarized; additional details may be found in previous reviews (45, 89–91).

1. **Metabolic Adaptation to Physiological Host Conditions**
  The fact that environmental cryptococci can infect many vertebrate and invertebrate hosts reflects its capacity to adapt to a variety of different conditions. Metabolic adaptation is a major requirement for fungal persistence in the mammalian host, and many genes and pathways that are essential for stress resistance and high temperature growth have been identified (90, 91). For example, the thermotolerant phenotype of *Cryptococcus sp*. is mediated by Ras1/Ras2 signaling pathways (92, 93) and functional calcineurin A, a Ca^2+^-calmodulin-regulated protein phosphatase that is activated by stress responses and stimulates the expression of genes required for growth and survival at 37°C as well as during oxidative stress (43, 89, 91, 94).
2. **Evasion and Interference With the Innate Immune Response**
  *Cryptococcus sp*. express several factors that have been shown to interfere with host immune response (72). For example, the extracellular capsule is a key virulence attribute that is composed of glucuronoxylomannan (GXM) and two minor components, galactoxylomannan (GalXMs), and mannoprotein (MP). The capsule conceals cell wall antigens, inhibits antibody binding to the fungal cell wall, activates and depletes complement, suppresses T lymphocyte proliferation, modulates cytokine production, and induces host cell apoptosis (95–97). Capsular enlargement during infection and formation of giant “Titan cells” that range in size from 50–100 μm is a powerful anti-phagocytic mechanism used by *Cryptococcus sp*. (98–100). Release of capsular GXM causes L-selectin shedding from neutrophils and limits their migration, adhesion to endothelial cells, and tissue extravasation (101). Cryptococcal capsular components also have anti-inflammatory properties that inhibit the maturation and activation of DCs, macrophages, and neutrophils (102– 104). Capsule-independent mechanisms including the App1 protein and GATA family of transcription factors have also been implicated in evasion of phagocytosis and immune recognition (105, 106).
  Several studies have shown long term survival of cryptococci within macrophages and endothelial cells during asymptomatic infection, suggesting that fungi may persist without causing tissue damage (72, 89). To survive within the harsh phagosomal environment, *Cryptococcus sp*. express several enzymes involved in nitric oxide detoxification and oxidative damage repair such as catalases, superoxide dismutases, glutathione peroxidases, thioredoxin proteins, the inositol phosphosphingolipid-phospholipase C1 (Isc1) and the protein kinase C (Pkc1) and utilize host lipid components for production of cryptococcal eicosanoids (107). Additional factors that promote intracellular survival and persistence include melanin, laccase, urease, phospholipase (PLB1) and heat shock protein 70 homolog Ssa1 (108–110).
  The ability to exit the phagocytic cells without killing and triggering an immediate immune response is one of the most important mechanisms associated with survival and long-term persistence of *Cryptococcus sp*. (10, 89). Non-lytic escape from phagocytes, also termed vomocytosis or phagosome extrusion, occurs by merging of the phagosome and plasma membranes followed by release of the organism to the surrounding environment or lateral transfer between host cells. Escape from phagocytes without triggering host cell death and inflammation is beneficial for latency and persistence of cryptococcal infection (84, 111). Finally, there is evidence that *Cryptococcus sp*. disseminates to the CNS from the bloodstream within macrophages using a Trojan Horse mechanism and is subsequently released by non-lytic extrusion after it has crossed the BBB (84, 111–114). Taken together, intracellular survival and non-lytic exocytosis are beneficial adaptations for both host and pathogen in the context of tolerance hypothesis (45).
3. **Interference With the Adaptive Immune Response**
  In addition to subversion of innate immunity, interference with the adaptive immune response is also essential for cryptococcal persistence and latent infection (89). *Cryptococcus sp*. use various mechanisms to regulate T-cell proliferation, differentiation, and survival (20, 115, 116). For example, expression of cryptococcal urease induces a non-protective Th2 immune response through recruitment of immature DCs to the lung-associated lymph nodes (117). Cleavage of fungal chitin by host chiotriosidase also initiates Th2 cell differentiation by CD11b^+^ conventional dendritic cells in pulmonary cryptococcal infection (118). Production of PGE2 by *C. neoformans* specifically inhibits IL-17 expression during Th17 cell differentiation in an IRF4-dependent manner (119). Inhibition of the Th17 response has been implicated as a potential mechanism that facilitates latent infection (89). Finally, persistent pulmonary *C. neoformans* infection also interferes with humoral immunity by selectively reducing antibody responses to exogenous cryptococcal polysaccharide (120).

## Host Immune Response Associated With Disease Tolerance in Cryptococcal Infection

Host resistance during cryptococcal infection is characterized by the expression of pro-inflammatory cytokines, recruitment of inflammatory DCs, and generation of Th1/Th17 immune responses that is followed by classical activation of macrophages (50, 51, 119, 121–126). However, excessive inflammation and robust Th1/Th17 responses that provide sterilizing immunity can induce severe pathology and damage to the host (59, 127–133). It has been proposed that a tightly regulated combination of pro-inflammatory and anti-inflammatory stimuli is crucial for effective control of fungal infection (134–136). In fact, immunoregulatory mechanisms that control the intensity and duration of the host response are one of the main strategies that may provide tolerance to infection and maintain host fitness and homeostasis (32, 33, 39). Below we describe cellular and molecular mechanisms that could mediate host tolerance during infection with *Cryptococcus sp*.

T-regulatory cells (Treg): Mutations in the Treg-associated transcription factor forkhead box protein P3 (FOXP3) are associated with development of severe immunopathology in both mice and humans, indicating that Tregs control tissue damage and contribute to disease tolerance (32). During fungal infection, activation of Treg cells is one of the critical mechanisms for reducing collateral damage to host tissues and restoring a homeostatic environment (66). Treg function is associated with production of the anti-inflammatory cytokines IL-10 and TGF-β that suppress the immune response (66, 135). In BALB/c mice, pulmonary CD4^+^ FoxP3^+^ Tregs increased during the first 4 weeks of infection with *C. neoformans* 1841. Conditional depletion of Tregs during the second week of infection, while both Th1 and Th2 responses were in progress, enhanced the Th2 response and suggested that Tregs limit Th2 cell proliferation and function in this model of infection (137). Another study demonstrated that the accumulation of antigen-specific Tregs in the *Cryptococcus*-infected lungs and their co-localization with Th2 effector cells occurs through expression of CCR5 and IFN regulatory factor 4 (IRF4) (138). In both reports, the immunoregulatory function of Tregs during acute cryptococcal infection was associated with reduced pathological Th2 responses; however, the possibility that long-term persistence of cryptococcal infection is also associated with an increase in Treg function remains to be investigated (66, 89).

IL-10 signaling: IL-10 is an anti-inflammatory cytokine expressed by Tregs and DCs that prevents excessive inflammation by limiting the production of IL-1, IL-6, IL-23, IFN-γ, and TNF-α during fungal infections (66, 135, 139). Early and sustained IL-10 production by lung leukocytes was demonstrated in a mouse model of persistent lung infection with *C. neoformans* 52D (140). C57BL/6 mice with genetically engineered IL-10 deficiency that were infected with *C. neoformans* demonstrated improved fungal clearance from the lung in association with reduced tissue eosinophilia, decreased expression of Th2 (IL-4, IL-5, and IL-13) and increased expression of Th1 (IL-12 and TNF-alpha) cytokines by lung leukocytes (141). Early or late interruption of IL-10 signaling after establishment of cryptococcal infection reduced fungal burden and dissemination to the brain and was associated with enhanced Th1/Th17 responses and increased activation and recruitment of CD11b^+^ DCs and exudate macrophages (140). In HIV+ patients with *C. neoformans* infection, a high level of IL-10 in the peripheral blood correlated with fungemia and dissemination (142). Therefore, the development of persistent or progressive cryptococcal infection appears to correlate with excessive IL-10 production while experimental IL-10 deficiency results in an enhanced inflammatory response (66).

DCs: Dendritic Cells (DCs) are the most efficient lineage for presentation of cryptococcal antigen to T cells and their activation is critical for activation of adaptive immunity that confers host protection. The role of DCs during cryptococcal infection has been recently reviewed (143, 144). The recruitment and maturation of DCs, as well as their ability to activate T cells, is affected by fungal characteristics as well as the local cytokine, chemokine, and scavenger receptor expression. Several soluble mediators including IL-4, IL-10, IL-17, and GM-CSF have been implicated in the recruitment, differentiation, and activation of DCs in different models of cryptococcal infection. Protection against *C. neoformans* is associated with recruitment and classical activation of monocyte-derived DCs (moDCs) resulting in secretion of pro-inflammatory cytokines and effective Th1/Th17 immune responses (145). Yet, moDCS are highly adaptable cells that can display inflammatory or immunoregulatory functions depending on the local cytokine microenvironment within infected tissues (66). For example, immunomodulatory or “tolerogenic” DCs can play an important role in regulation of inflammation and immunopathology through secretion of anti-inflammatory cytokines, induction of hyporesponsiveness, and promotion of peripheral or induced Treg cells (146). Human and murine monocytes and DCs that were stimulated *in vitro* with *C. neoformans* antigen produced a significant amount of the immunomodulatory cytokine IL-10 (102, 147). In addition, development of immunomodulatory DCs in a murine model of persistent *C. neoformans* infection was associated with Th1 and Th17 suppression, reduced macrophage activation, and impaired fungal clearance (66, 140, 141).

Tryptophan pathway: Indoleamine 2,3-dioxygenase (IDO), a metabolic enzyme involved in tryptophan degradation and production of kynurenines, plays an important role in the balance between Tregs and Th1/Th17 cells (148, 149). Expression of IDO by DCs results in a tolerogenic phenotype that is associated with immune homeostasis, suppression of inflammation and effector T cells, induction of Tregs, and enhanced tolerance to fungal infection at mucosal surfaces (134, 135, 150, 151). The expression of IDO by host cells following cryptococcal infection has not been reported and could be a potential mechanism of disease tolerance.

Fas-associated death domain (FADD) and receptor interacting protein kinase 3 (RIPK3): The FADD protein is a key mediator of death receptor-triggered extrinsic apoptosis, which plays a crucial immune regulatory role at the site of infection and prevents excessive inflammation (127). Deletion of RIPK3 in combination with FADD led to a robust Th1-biased response with M1-biased macrophage activation, yet this host response was deleterious in a mouse model of cryptococcal infection. The excessive mortality in RIPK3 or RIPK3/FADD knockout mice was associated with significant pulmonary damage due to neutrophil-dominant infiltration with marked upregulation of pro-inflammatory cytokines. These findings demonstrate the role of both molecules in protection of the host by limiting excessive inflammation and conferring tolerance during cryptococcal infection (127).

T cell exhaustion: The loss of proliferation and limited effector function of T cells during states of chronic infection could be viewed as a tolerance-associated mechanism (152). Multiple pathways may mediate a state of T cell exhaustion; for example, binding of Cytotoxic T Lymphocyte-Associated Protein 4 (CTLA4) to co-stimulatory molecules CD80 and CD86 blocks CD28-mediated T cell co-stimulation and inhibits T cell activation and function (151). *C. neoformans* has been shown to rapidly induce CTLA-4 upregulation on murine CD4^+^ T cells (153). Blockade of CTLA-4 on *C. neoformans*-stimulated CD4^+^ T cells resulted in enhanced proliferation and IL-2/IFN-γ cytokine production. In addition, differential CTLA-4 upregulation was observed when cells were stimulated with an encapsulated strain of *C. neoformans*. In another study CTLA-4 blockade enhanced fungal control and survival of mice that were subsequently infected with highly virulent *C. neoformans* (154). These results indicate that the induction of CTLA-4 could be a mechanism used by cryptococci to diminish the immune response and facilitate persistent infection (66). Similarly, the contribution of the programmed cell death protein-1 (PD-1) during cryptococcal infection in C57BL/6 mice has been investigated (155). The results demonstrated an association between persistent infection and increased and sustained expression of PD-1 on CD4^+^ T cells as well as upregulation of PD-1 ligands on specific subsets of resident and recruited DCs and macrophages. Furthermore, PD-1 blockade significantly improved pulmonary fungal clearance. Based on current data, the role of CTLA-4 and PD-1 as potential mediators of disease tolerance could be further studied, for example, in the context of cryptococcal IRIS. In conclusion, protective tolerance during persistent cryptococcal infection has been associated with the development of immunomodulatory/tolerogenic DCs and expression of IL-10, IDO, CTL4 and PD-1 (66).

## Conclusion And Future Directions

In states of persistent cryptococcal infection, a tightly-regulated balance between resistance and tolerance mechanisms is required to maintain host fitness and homeostasis. Several lines of evidence indicate that *C. neoformans* plays an important role in maintaining host tolerance to favor their own survival. The ability to survive within mammalian cells and to subvert or evade the host immune response without causing damage may be the inadvertent consequences of a long evolutionary path taken by this free environmental yeast to adapt to ecological selection pressures. Within the context of the damage response framework, infection of the host by a microbe is not a major concern in the absence of significant damage. Therefore, in latent cryptococcal infection one might postulate that the fungus is no longer considered to be a pathogen by the host immune system (47, 66).

Morbidity and mortality in cryptococcal infection can result from defective host resistance in advanced states of immunodeficiency, or a failure of tolerance mechanisms as observed during cIRIS. As the spectrum of hosts with cryptococcal disease expands, the ability to understand and distinguish tolerance-associated mechanisms from failures of host resistance will have important therapeutic implications. For example, bolstering immunity to further reduce pathogen burden may be unsuccessful in cases of defective tolerance with significant tissue and/or organ damage, while immunomodulation may be beneficial (31, 52, 156). Thus, a comprehensive therapeutic strategy that takes host resistance and tolerance mechanisms into account could have potential to significantly improve disease outcomes (157–159).

## Author Contributions

MS wrote and edited the manuscript. SQ edited the manuscript and revised it critically for important intellectual content.

### Conflict of Interest Statement

The authors declare that the research was conducted in the absence of any commercial or financial relationships that could be construed as a potential conflict of interest. The handling Editor declared a shared affiliation, though no other collaboration, with the authors SQ and MS.
